# Supplementary material for: The Genomics of Streptococcus pneumoniae Carriage Isolates from UK Children and Their Household Contacts, Pre-PCV7 to Post-PCV13
Source: Genes (Basel). 2019 Sep 6;10(9):687. doi: 10.3390/genes10090687 (PMC6771020; doi:10.3390/genes10090687)
Supplement: Supplementary file 1 [file genes-10-00687-s001.zip › genes-582961-supplmentary/Supplementary Table 5_roary_stats.docx]

**Supplementary Table S5.** Gene presence summary statistics from Roary analysis.

| **Type** | **Number of genes** |
| --- | --- |
| Core genes (Present in >99% strains) | 1350 |
| Soft core genes (Present+ in between 95-99% strains) | 181 |
| Shell genes (Present in between 15-95% strains) | 701 |
| Cloud genes (Present in between 0-15% strains) | 6321 |
| Total genes | 8553 |
