# Supplementary material for: The Genomics of Streptococcus pneumoniae Carriage Isolates from UK Children and Their Household Contacts, Pre-PCV7 to Post-PCV13
Source: Genes (Basel). 2019 Sep 6;10(9):687. doi: 10.3390/genes10090687 (PMC6771020; doi:10.3390/genes10090687)
Supplement: Supplementary file 1 [file genes-10-00687-s001.zip › genes-582961-supplmentary/Supplementary_Table_3_GPSC_with_multiple_Local_clusters.docx]

Supplementary Table S3. GPSC associated with more than one local dataset PopPUNK cluster.

| GPSC cluster | Local dataset cluster |
| --- | --- |
| 3 | 2, 23, 39, 48 |
| 7 | 1, 6 |
| 11 | 8, 15, 40 |
| 12 | 27, 46 |
| 13 | 49, 73, 79 |
| 16 | 30, 47, 52, 74 |
| 18 | 13, 41, 66 |
| 36 | 12, 20 |
| 45 | 42, 53 |
| 57 | 22, 72 |
| 72 | 34, 63 |
| 76 | 56, 77 |
| 316 | 33, 76 |
